# Clinical features associated with occult pneumonia among older adults with community-acquired pneumonia: a descriptive retrospective study

**DOI:** 10.3389/fmed.2026.1966431

**Published:** 2026-10-21

**Authors:** Liu Panpan, Lei Manman, Qin Fufang

**Affiliations:** Department of General Practice, The Fifth Clinical Medical College of Henan University of Traditional Chinese Medicine (Zhengzhou People's Hospital), Zhengzhou, China

**Keywords:** occult pneumonia, older adults, community-acquired pneumonia, associated factors, diabetes mellitus, cerebrovascular disease

## Abstract

**Background:**

Pneumonia is a major global health burden and a substantial threat to older adults. Because pneumonia in this population may present insidiously without typical respiratory symptoms, occult pneumonia can be easily overlooked, resulting in delayed treatment and an increased risk of severe disease and death. This study aimed to identify clinical features associated with occult pneumonia.

**Methods:**

We retrospectively reviewed the medical records of 334 older adults with pulmonary infections admitted to our hospital between January 2023 and January 2025. Patients were randomly assigned to a development cohort (*n* = 234) and an internal validation cohort (*n* = 100) at a 7:3 ratio. The development cohort comprised 80 patients with occult pneumonia and 154 with typical pneumonia. Univariate and multivariable logistic regression analyses were performed to identify factors independently associated with occult pneumonia.

**Results:**

LASSO logistic regression with 5-fold cross-validation and backward-stepwise logistic regression identified five independent predictors of occult pneumonia: age ≥80 years (OR = 2.584, 95% CI: 1.350–4.948), diabetes mellitus (OR = 3.392, 95% CI: 1.696–6.782), cerebrovascular disease (OR = 2.116, 95% CI: 1.027–4.357), decreased appetite (OR = 4.982, 95% CI: 2.609–9.513), and WBC count (OR = 2.797, 95% CI: 1.290–6.067; all *P* < 0.05). The association with WBC count was positive and graded: WBC counts were higher in patients with occult than typical pneumonia (median 6.92 vs. 6.06  ×  10⁹/L, *P* = 0.002), and in sensitivity analyses each 1 × 10⁹/L increase in WBC count was associated with an 18% increase in the odds of occult pneumonia (OR = 1.184, 95% CI: 1.051–1.334) without evidence of non-linearity (*P* = 0.920). The model achieved AUCs of 0.799 (95% CI: 0.739–0.854) in the development cohort and 0.799 (95% CI: 0.699–0.886) in the internal validation cohort. Calibration was assessed using the calibration intercept, slope, and Brier score, which indicated good agreement between predicted and observed probabilities.

**Conclusion:**

Age ≥80 years, diabetes mellitus, cerebrovascular disease, decreased appetite, and WBC count were independently associated with occult pneumonia in older adults. These factors characterize the occult presentation phenotype and may help clinicians recognize occult pneumonia among older adults with CAP to support appropriate evaluation and management.

## 1 Introduction

Pneumonia is a common acute respiratory infection and remains a major contributor to the global burden of infectious diseases. According to publicly available data from the 2019 Global Burden of Disease (GBD) Study, lower respiratory infections (LRIs) caused approximately 489 million cases and 2.4 million deaths worldwide in 2019. Mortality was particularly high among children and older adults, underscoring the disproportionate impact of LRIs on vulnerable populations (1). Moreover, a related analysis based on the 2023 GBD Study showed that LRIs continued to rank among the leading causes of disability-adjusted life years (DALYs) and death across age groups, highlighting their importance as a major public health challenge and a frequent cause of severe infection in clinical practice (2, 3). Community-acquired pneumonia (CAP) is the most common form of pneumonia encountered in clinical practice and accounts for a substantial proportion of the overall pneumonia burden. In most middle- and high-income countries, CAP is among the leading infectious causes of hospitalization in adults and is a major contributor to infection-related mortality (4, 5). In the United States, CAP is one of the most common infectious causes of hospitalization and death among adults. In 2019, it was estimated to account for approximately 740,000 hospitalizations and 41,000 deaths annually, with hospitalization-related costs reaching $7.7 billion, thereby imposing a substantial socioeconomic burden (6). In China, the incidence of CAP was estimated at 7.13 cases per 1,000 person-years, and more than 9.5 million cases were reported in 2016. With population aging and increasing life expectancy, the number of older adults in China continues to grow. Consequently, the incidence of CAP and the number of CAP-related deaths are expected to increase, further intensifying the disease burden in China (7).

Older adults are particularly susceptible to pneumonia because of age-related immune senescence, diminished physiological reserve, and the high prevalence of comorbidities (8, 9). Clinically, pneumonia in older adults often presents without typical respiratory symptoms, and this atypical presentation is especially common among the oldest-old. The onset is frequently insidious, with early manifestations dominated by nonspecific symptoms such as fatigue, decreased appetite, and altered mental status (10, 11). For the purposes of this study, occult pneumonia refers to pneumonia presenting with atypical or minimal respiratory symptoms that may be overlooked during the initial clinical evaluation. Traditional inflammatory biomarkers, including the white blood cell (WBC) count, high-sensitivity C-reactive protein (hs-CRP), and procalcitonin (PCT), may have limited sensitivity in older adults and can be affected by underlying diseases and other comorbid conditions (12, 13). Consequently, these biomarkers may not provide definitive evidence of infection. Older adults with CAP are therefore at increased risk of delayed or missed diagnosis. Such delays may increase the risk of progression to severe disease and death and may result in inappropriate antibiotic use or failure to initiate treatment within the optimal therapeutic window. Currently, evidence regarding occult pneumonia remains limited both nationally and internationally, and no universally accepted definition or diagnostic criteria have been established. This lack of consensus presents substantial challenges for early clinical recognition and standardized management. Because the clinical manifestations are often atypical and difficult to detect during the early stages, occult pneumonia may not be recognized until the disease has progressed to severe pneumonia, sepsis, or respiratory failure. This progression can prolong hospitalization, increase healthcare costs, and adversely affect patient outcomes (14, 15). Accordingly, occult pneumonia warrants greater attention in clinical practice.

This study focuses on occult pneumonia in older adults, a clinically important high-risk presentation. Using a retrospective analysis of medical records from older adults with pneumonia, we aimed to identify clinical features associated with occult pneumonia and to characterize the factors independently associated with this presentation. The findings may help clinicians recognize occult pneumonia among older adults with CAP and support appropriate evaluation.

## 2 Methods

### 2.1 Study design

This was a single-center, retrospective observational study. Clinical data were consecutively collected from patients who were hospitalized at our hospital between January 2023 and January 2025 and had a confirmed diagnosis of CAP in older adults at discharge. After screening, 334 eligible patients were included and randomly assigned to a development cohort (*n* = 234) and an internal validation cohort (*n* = 100) at a ratio of 7:3 using a random-number table. Patients in the development cohort were further classified according to the presence or absence of typical clinical manifestations into an occult pneumonia group (*n* = 80) and a typical pneumonia group (*n* = 154). The development cohort was used to derive the multivariable model, whereas the internal validation cohort was used to evaluate its stability. The study protocol was reviewed and approved by the Ethics Committee of the Zhengzhou People's Hospital, and the requirement for informed consent was waived. Because of the retrospective observational design, only previously recorded medical data were analyzed, and no additional interventions were performed. All study procedures were conducted in accordance with the Declaration of Helsinki and applicable ethical standards.

### 2.2 Case selection

#### 2.2.1 Diagnostic criteria

The study population comprised patients aged 60 years or older who were hospitalized at our hospital between January 2023 and January 2025 and received a confirmed diagnosis of CAP at discharge. CAP was diagnosed in accordance with the Chinese Guidelines for the Diagnosis and Treatment of Community-Acquired Pneumonia in Adults (2016 Edition) (16). The specific diagnostic criteria were as follows: 1.Community onset; 2.Pneumonia-related clinical manifestations: a. Newly developed cough or sputum production, or worsening of pre-existing respiratory symptoms, with or without purulent sputum, chest pain, dyspnea, or hemoptysis; b. Fever; c. Signs of pulmonary consolidation and/or moist crackles on auscultation; d. Peripheral blood leukocyte count >10 × 10⁹/L or <4 × 10⁹/L, with or without a left shift; 3.Chest imaging showing newly developed patchy infiltrates, lobar or segmental consolidation, ground-glass opacities, or interstitial changes, with or without pleural effusion. A clinical diagnosis can be established when criteria 1 and 3 are met, together with any one of items a to d under criterion 2, after excluding pulmonary tuberculosis, lung tumors, noninfectious interstitial lung disease, pulmonary edema, atelectasis, pulmonary embolism, pulmonary eosinophilic infiltration, pulmonary vasculitis, and other relevant conditions.

#### 2.2.2 Inclusion criteria

Patients were included if they met all of the following criteria:

1. Age ≥60 years;
2. Fulfillment of the clinical diagnostic criteria for CAP;
3. Actual length of hospital stay >3 days;
4. Clinical data fully available

#### 2.2.3 Exclusion criteria

Patients meeting any of the following criteria were excluded:

1. Critical illness associated with a high risk of short-term death or an immediate need for life-support therapy after admission;
2. Severe immunosuppression, including hematologic disease, acquired immunodeficiency syndrome, or a history of bone marrow transplantation;
3. End-stage chronic disease;
4. Hospital-acquired pneumonia (HAP), defined as pneumonia that was not present at admission and developed 48 h or more after hospital admission (17);
5. Neutropenia or ongoing radiotherapy or chemotherapy;
6. Psychiatric disorders that could affect the assessment of clinical symptoms or the reliability of the clinical data; the detailed eligibility criteria are summarized in Table 1.

**Table 1:** Inclusion and exclusion criteria.

Type	Criterion
Inclusion	Age ≥60 years
Inclusion	Fulfillment of the clinical diagnostic criteria for community-acquired pneumonia (CAP)
Inclusion	Actual length of hospital stay >3 days
Inclusion	Clinical data fully available
Exclusion	Critical illness with high risk of short-term death or immediate need for life-support therapy after admission
Exclusion	Severe immunosuppression, including hematologic disease, acquired immunodeficiency syndrome, or a history of bone marrow transplantation
Exclusion	End-stage chronic disease
Exclusion	Hospital-acquired pneumonia (HAP), defined as pneumonia that was not present at admission and developed 48 h or more after hospital admission; this definition follows the ATS/IDSA hospital-acquired pneumonia guideline (17).
Exclusion	Neutropenia or ongoing radiotherapy or chemotherapy
Exclusion	Psychiatric disorders that could affect the assessment of clinical symptoms or the reliability of the clinical data

### 2.3 Grouping criteria

Patients in the development cohort were classified into the occult pneumonia group or the typical pneumonia group according to their clinical manifestations and ancillary examination findings. Typical pneumonia was defined as CAP accompanied by typical respiratory symptoms, such as cough, sputum production, or chest pain, with the diagnosis confirmed by chest imaging and other relevant clinical findings. Occult pneumonia was defined according to previously published criteria (14, 18, 19). The specific diagnostic criteria were as follows: (1) The patient had no typical respiratory symptoms, including cough, sputum production, or chest pain; (2) Chest computed tomography (CT) showed imaging findings consistent with pneumonia; (3) When chest CT showed no obvious abnormalities or could not be performed because of the patient's clinical condition or difficulties with transportation, at least one of the following criteria was also required: a positive culture of an adequate sputum specimen and/or blood culture; elevated infection-related biomarkers, limited to procalcitonin (PCT) and high-sensitivity C-reactive protein (hs-CRP); or a favorable response to empirical anti-infective therapy. The detailed criteria used to distinguish occult pneumonia from typical pneumonia are provided in Table 2.

**Table 2:** Comparison of typical and occult presentation among patients with confirmed community-acquired pneumonia (CAP).

Dimension	Typical CAP	Occult CAP
Basic diagnostic requirement	A confirmed diagnosis of community-acquired pneumonia (CAP)	A confirmed diagnosis of CAP
Typical respiratory symptoms	Present, including one or more typical respiratory symptoms, such as cough, sputum production, or chest pain	Absent, specifically no cough, sputum production, or chest pain
Chest CT findings	Chest imaging findings consistent with pneumonia, together with other relevant clinical findings	Chest CT findings consistent with pneumonia
Additional diagnostic evidence	Peripheral blood leukocyte count >10 × 10⁹/L or <4 × 10⁹/L, with or without a left shift.	At least one of the following: positive sputum and/or blood culture, elevated PCT and/or hs-CRP levels, or a favorable response to empirical anti-infective therapy

### 2.4 Data collection

Clinical data were retrieved from the hospital information system and the medical-record management system using a prespecified standardized data-collection form. The collected variables included demographic characteristics, underlying diseases and comorbidities, clinical manifestations at admission, and laboratory test results.

1. Demographic characteristics: age, sex, smoking status, prolonged bed rest, and residence in a nursing home or long-term care facility;
2. Underlying diseases and comorbidities: diabetes mellitus, coronary artery disease, congestive heart failure, cerebrovascular disease, chronic obstructive pulmonary disease, hepatic or renal insufficiency, malnutrition, and other relevant diseases.
3. Clinical manifestations at admission: fever, cough, sputum production, chest tightness, dyspnea, fatigue, decreased appetite, and altered mental status;
4. Laboratory tests: complete blood count, albumin(Alb), PCT, hs-CRP, liver function tests, and renal function tests.

All data were independently extracted and entered by two researchers who had received standardized training. After data entry, the records were cross-checked to verify the accuracy and completeness of the dataset.

### 2.5 Statistical analysis

Statistical analyses were performed using SPSS version 27.0 (IBM Corp., Armonk, NY, USA). Continuous variables with a normal distribution were expressed as the mea*n* ± standard deviation and compared using the independent-samples t test. Non-normally distributed continuous variables were expressed as the median and interquartile range and compared using the Mann–Whitney U test. Categorical variables were summarized as frequencies and percentages and compared using the chi-square test or Fisher's exact test, as appropriate. Predictor selection was performed strictly within a pre-specified candidate set. Before any modelling we defined 14 candidate predictors *a priori* on the basis of published evidence and clinical plausibility for the occult/atypical presentation of pneumonia in older adults. All candidates were captured by the pre-specified standardized data-collection form and were available before analysis; univariable significance was not used to decide candidacy. Within this set, independent predictors were selected using backward stepwise multivariable logistic regression; LASSO (L1-penalized) logistic regression with 5-fold cross-validated regularization was used as a sensitivity check for the selection; at the cross-validation-selected penalty, LASSO retained all five predictors selected by the stepwise procedure together with three additional candidates (residence in a nursing home, prolonged bed rest, and procalcitonin), and the more parsimonious five-predictor set was carried forward as the final model to identify independent predictors of occult pneumonia. Multicollinearity among the candidate predictors was assessed using variance inflation factors (VIF), and predictors with VIF > 5 were addressed by retaining the most clinically informative marker; because altered mental status showed complete separation (present in 9 occult vs. 0 typical patients), a Firth penalized logistic regression was performed as a sensitivity analysis to confirm the direction and magnitude of the retained predictors. A multivariable logistic regression model was fitted using Python (version 3.13) with the statsmodels library. Receiver operating characteristic (ROC) curves and the area under the ROC curve (AUC) were used to evaluate the model's discrimination. Calibration performance was assessed using calibration curves and quantified by the calibration intercept, slope, and Brier score (replacing the Hosmer–Lemeshow goodness-of-fit test). Model performance was assessed in both the development and internal validation cohorts. To correct for optimism introduced by the single random split, bootstrap optimism correction (B = 200 resamples) was performed on the development cohort; optimism-corrected AUC, Somers' Dxy, calibration slope and intercept, and Brier score are reported below. This study adheres to the TRIPOD + AI reporting guideline for clinical prediction model development and validation; the completed TRIPOD + AI checklist is provided as a supplementary file. All statistical and modelling analyses were performed in Python (version 3.13) using the pandas, NumPy, statsmodels, and scikit-learn libraries.

## 3 Results

### 3.1 Univariate analysis of factors associated with occult pneumonia

#### 3.1.1 Comparison of general clinical characteristics between the occult pneumonia and typical pneumonia groups

A total of 407 older patients with pulmonary infection who were admitted to our hospital between January 2023 and January 2025 were initially screened. Of these, 46 were excluded because of incomplete medical records, 2 because of a hospital stay of ≤3 days, and 25 because of duplicate hospitalizations. Ultimately, 334 patients were included in the analysis. Of these, 234 were assigned to the development cohort, including 80 patients with occult pneumonia and 154 with typical pneumonia, and 100 were assigned to the internal validation cohort. A STROBE-compliant flow diagram of patient inclusion and exclusion is provided in Supplementary Figure S1.Univariate analysis showed significant differences between the occult pneumonia and typical pneumonia groups in age, sex, prolonged bed rest, cerebrovascular disease, and diabetes mellitus (all *P* *<* *0.05*). No significant differences were observed in residence in a nursing home or long-term care facility, smoking history, chronic obstructive pulmonary disease, malnutrition, coronary artery disease, or hepatic or renal insufficiency (all *P* *>* *0.05*). The detailed results are presented in Table 3.

**Table 3:** General clinical characteristics.

Variable	occult pneumonia group（*n* = 80)	typical pneumonia group（*n* = 154)	*χ^2^/t* value	*P* value
Age, *n* (%)			12.348	＜0.001
≥80 years	45（56.25）	50（32.47）		
<80 years	35（43.75）	104（67.53）		
Gender, *n* (%)			4.673	0.031
Male	43（53.75）	60（38.96）		
Female	37（46.25）	94（61.04）		
residence in a nursing home, *n* (%)	1（1.25）	0（0.00）	—	0.342
prolonged bed rest, *n* (%)	11（13.75）	7（4.55）	6.282	0.012
smoking history, *n* (%)	6（7.50）	9（5.84）	0.241	0.624
Comorbidities, *n* (%)				
coronary artery disease	30（37.50）	50（32.47）	0.593	0.441
cerebrovascular disease	32（40.00）	26（16.88）	15.092	＜0.001
hepatic or renal insufficiency	6（7.50）	6（3.90）	0.762	0.383
chronic obstructive pulmonary disease (COPD)	1（1.25）	3（1.95）	—	1.000
malnutrition	0（0.00）	1（0.65）	—	1.000
diabetes mellitus	36（45.00）	30（19.48）	16.932	＜0.001

#### 3.1.2 Comparison of clinical symptoms between the two groups

Univariate analysis showed significant differences between the occult pneumonia and typical pneumonia groups in fatigue, decreased appetite, and altered mental status (all *P* *<* *0.05*). No significant differences were observed in chest tightness or dyspnea (all *P* *>* *0.05*). The detailed results are presented in Table 4.

**Table 4:** clinical symptoms.

Variable	occult pneumonia group（*n* = 80)	typical pneumonia group（*n* = 154)	*χ^2^* value	*P* value
fever, *n* (%)	44（55.00）	94（61.04）	0.794	0.373
chest tightness, *n* (%)	26（32.50）	65（42.21）	2.088	0.148
dyspnea, *n* (%)	1（1.25）	10（6.49）	2.167	0.141
fatigue, *n* (%)	41（51.25）	47（30.52）	9.643	0.002
decreased appetite, *n* (%)	51（63.75）	41（26.62）	30.417	＜0.001
altered mental status, *n* (%)	9（11.25）	0（0.00）	15.104	＜0.001

#### 3.1.3 Comparison of laboratory test results between the two groups

Univariate analysis showed significant differences between the occult pneumonia and typical pneumonia groups in WBC count, neutrophil count, lymphocyte count, Alb, PCT, hs-CRP, creatinine (Cr), and blood urea nitrogen (BUN) levels (all *P* *<* *0.05*). No significant differences were observed in red blood cell (RBC) count, hemoglobin concentration (Hb), or platelet (PLT) count (all *P* *>* *0.05*). The detailed results are presented in Table 5.

**Table 5:** laboratory tests.

Variable	occult pneumonia group（*n* = 80)	typical pneumonia group（*n* = 154)	*Z*/*t* value	*P* value
RBC(×10^12^/L)	3.96 ± 0.66	4.09 ± 0.51	−1.571^a^	0.119
Hb(g/L)	120.49 ± 19.62	125.05 ± 15.98	−1.911^a^	0.057
Alb(g/L)	35.85 ± 4.88	37.57 ± 4.27	−2.794^a^	0.006
WBC(×10^9^/L)	6.92（5.40，9.95）	6.06（4.84，7.37）	−3.052	0.002
neutrophil count (×10^9^/L)	5.41（3.69，8.14）	4.03（3.18，5.42）	−3.765	＜0.001
lymphocyte count(×10^9^/L)	1.04（0.64，1.43）	1.17（0.81，1.69）	−2.065	0.039
PLT(×10^9^/L)	199.50（148.25，255.75）	196.00（154.75，248.50）	−0.338	0.735
PCT(ng/mL)	0.085（0.043，0.184）	0.062（0.038，0.091）	−2.530	0.011
hs-CRP(mg/L)	21.98（3.62，58.52）	9.82（1.93，38.07）	−2.219	0.027
Cr(µmol/L)	74.00（58.00，97.00）	67.00（56.00，82.00）	−2.502	0.012
BUN(mmol/L)	6.24（4.65，8.59）	5.21（4.17，6.29）	−3.779	＜0.001

RBC, red blood cell count; Hb,: hemoglobin concentration; Alb, albumin; WBC, white blood cell count; PLT, platelet count; PCT, procalcitonin; hs-CRP, high-sensitivity C-reactive protein; Cr, creatinine; BUN, blood urea nitrogen.

### 3.2 Multivariate analysis of factors associated with occult pneumonia

Within a pre-specified candidate set of 14 predictors defined *a priori* from the literature and clinical plausibility for occult/atypical pneumonia in older adults, variable selection was performed using L1 (LASSO) penalized logistic regression, with the penalty (lambda) chosen by 5-fold cross-validation; predictors with nonzero shrunken coefficients were retained. For transparency, a conventional backward-stepwise logistic regression within the same pre-specified set was also performed and selected the identical five predictors; LASSO retained these five together with three additional candidates (residence in a nursing home, prolonged bed rest, and procalcitonin), supporting selection stability (see Figure 1) (20). Variables were coded as follows: the presence of occult pneumonia was coded as 1, whereas typical pneumonia was coded as 0; age <80 years was coded as 0 and age ≥80 years as 1; the presence of fatigue, decreased appetite, diabetes mellitus, or cerebrovascular disease was coded as 1, whereas its absence was coded as 0. WBC count was categorized as <3.5, 3.5–9.5, and >9.5 × 10⁹/L and assigned category codes of 1, 2, and 3, respectively. Multivariable logistic regression analysis identified age ≥80 years (OR = 2.584, 95% CI: 1.350–4.948), diabetes mellitus (OR = 3.392, 95% CI: 1.696–6.782), cerebrovascular disease (OR = 2.116, 95% CI: 1.027–4.357), decreased appetite (OR = 4.982, 95% CI: 2.609–9.513), and WBC count (OR = 2.797, 95% CI: 1.290–6.067) as independent factors associated with occult pneumonia (all *P* < 0.05). The model intercept was −4.5618 (SE = 0.9410), representing the log-odds of an occult presentation at the reference level of all predictors; this intercept is required to compute individual probabilities from the model. The detailed results are presented in Table 6. The association with WBC count was positive and graded: patients with occult pneumonia had higher WBC counts than those with typical pneumonia (median 6.92 [interquartile range 5.40–9.95] vs. 6.06 [4.84–7.37] × 10⁹/L; *P* = 0.002; Table 5), and in the final model, in which WBC count entered as an ordinal variable (<3.5, 3.5–9.5, and >9.5 × 10⁹/L), each one-band increase was associated with a 2.797-fold increase in the odds of occult pneumonia, indicating a graded rather than a threshold relationship. In a sensitivity analysis, WBC count was additionally modelled as a continuous linear predictor and as a restricted cubic spline (3 degrees of freedom) to evaluate the functional form of its association with occult pneumonia; the spline was compared with the linear form using a likelihood-ratio test; confidence bands for the spline-based dose–response curve were obtained by bootstrap resampling (B = 300; Figure 2).

**Figure 1:**
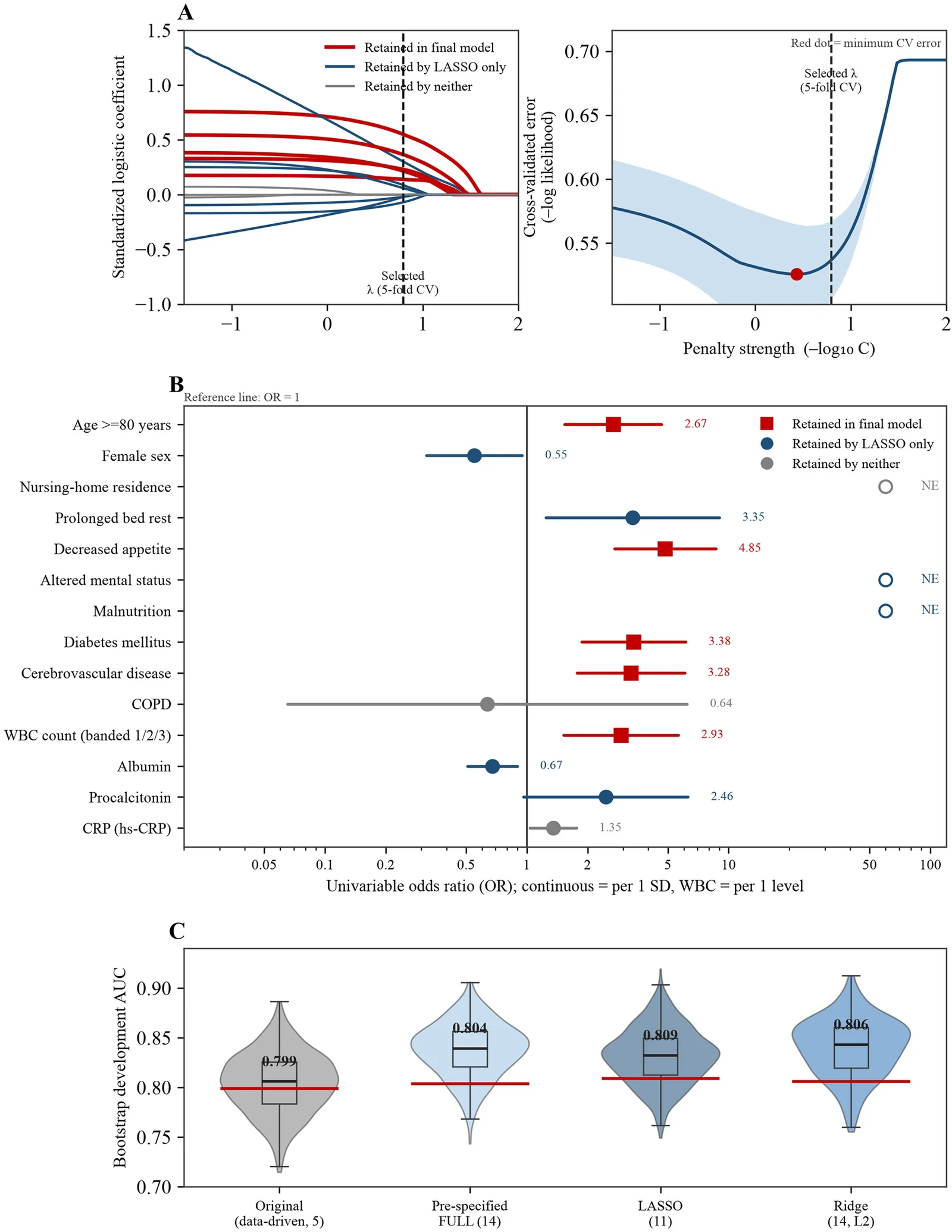
Pre-specified candidate predictor selection and model stability. **(A)** LASSO (L1) penalized selection within the pre-specified candidate set, shown as the coefficient path (left: standardized logistic coefficients versus penalty strength) and the 5-fold cross-validation error (right: mean −log likelihood ± 1 SE, red dot = minimum); the five predictors retained in the final model are shown in red and those retained by LASSO only in blue, and the cross-validation-selected penalty is marked by the dashed vertical line — the stepwise procedure selected five predictors, all of which were also retained by LASSO, which additionally retained three candidates (residence in a nursing home, prolonged bed rest, and procalcitonin) at the selected penalty. **(B)** Univariable odds ratios (log scale) with 95% confidence intervals for each candidate predictor in the development cohort; continuous variables are expressed per 1 SD and WBC count (banded) per 1 level. Red squares denote predictors retained in the final model, blue circles denote predictors retained by LASSO only, and grey circles denote predictors retained by neither; NE indicates an odds ratio not estimable owing to complete or quasi-complete separation. **(C)** Bootstrap distribution (violin and box; 1,000 resamples) of the development AUC for the original data-driven model, the full pre-specified model, and the LASSO- and Ridge-penalized models; the red dashed line marks the internal-validation AUC of each model.

**Table 6:** Multivariate logistic regression analysis of features associated with occult pneumonia.

Variable	*β*	SE	Wald value	*P* value	OR	95%CI
Age ≥80 years	0.949	0.331	8.205	0.004	2.584	1.350∼4.948
diabetes mellitus	1.221	0.354	11.937	＜0.001	3.392	1.696∼6.782
cerebrovascular disease	0.749	0.369	4.134	0.042	2.116	1.027∼4.357
decreased appetite	1.606	0.330	23.673	＜0.001	4.982	2.609∼9.513
WBC count*	1.029	0.395	6.779	0.009	2.797	1.290∼6.067
Intercept	−4.562	0.941	—	—	0.010	0.002∼0.066

WBC count*, white blood cell count was categorized as <3.5, 3.5–9.5, and >9.5 × 10⁹/L; the OR represents the change in odds associated with a one-category increase.

**Figure 2:**
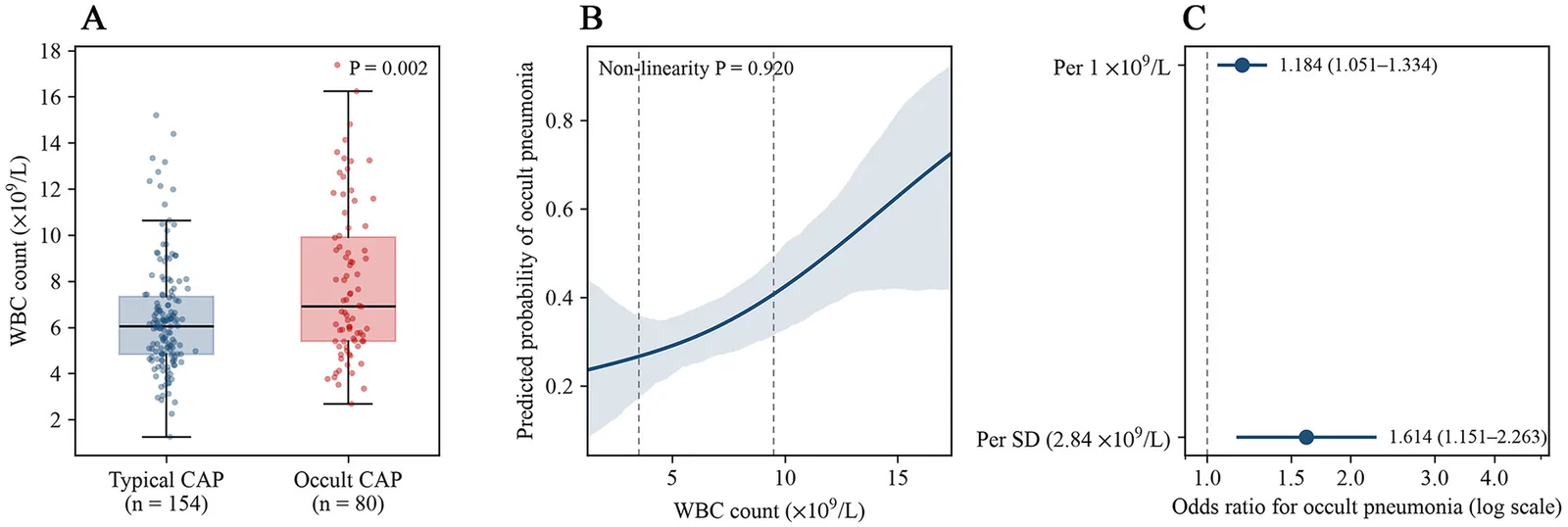
Quantitative analysis of WBC count and occult pneumonia. **(A)** WBC count by group (boxes, median and interquartile range; points, individual patients; *P* value from the Mann–Whitney U test). **(B)** Cohort-averaged dose–response association between continuous WBC count and the predicted probability of occult pneumonia from a restricted cubic spline (3 degrees of freedom), adjusted for age, diabetes mellitus, cerebrovascular disease, and decreased appetite; shaded band, bootstrap 95% confidence interval (B = 300); dashed vertical lines, the 3.5 and 9.5 × 10⁹/L cut-points of the ordinal coding; *P* value from the likelihood-ratio test of non-linearity. **(C)** Odds ratios for occult pneumonia per 1 × 10⁹/L and per standard deviation (2.84 × 10⁹/L) increase in WBC count from the continuous linear model; error bars, 95% confidence intervals.

### 3.3 Performance of the multivariable model in the development and internal validation cohorts

Based on the multivariable logistic regression model, the performance of the identified independent predictors was evaluated. The model was validated using the ROC curve and the area under the curve (AUC). The results showed that the AUC for the development cohort was 0.799 (95% CI: 0.739–0.854) and for the internal validation cohort was 0.799 (95% CI: 0.699–0.886), indicating good discriminative performance of the model (see Figure 3). ROC curves were constructed from the predicted probabilities of the five-predictor multivariable model rather than from WBC levels alone; no single WBC cutoff was used for model evaluation, and WBC count alone showed lower discrimination (AUC 0.593 in the development cohort and 0.526 in the internal validation cohort). Calibration was quantified using the calibration intercept, slope, and Brier score. In the development cohort the intercept was 0.00 (ideal = 0), the slope was 1.000, and the Brier score was 0.163; in the internal validation cohort the intercept was 0.178, the slope was 1.185, and the Brier score was 0.164, indicating good agreement between predicted and observed probabilities. Bootstrap optimism correction (B = 200) on the development cohort indicated only modest optimism: the optimism-corrected AUC was 0.788 (corrected Somers' Dxy 0.577), the calibration slope was 0.925 (ideal 1.000), the calibration intercept was −0.028 (ideal 0.000), and the corrected Brier score was 0.172. The calibration curve in Figure 4A represents the bootstrap-corrected estimate. Additionally, the calibration curves for both the development and internal validation cohorts closely approximated the ideal curve, suggesting that the model's predicted probability of occult pneumonia occurrence aligns well with the actual probability (see Figure 4).

**Figure 3:**
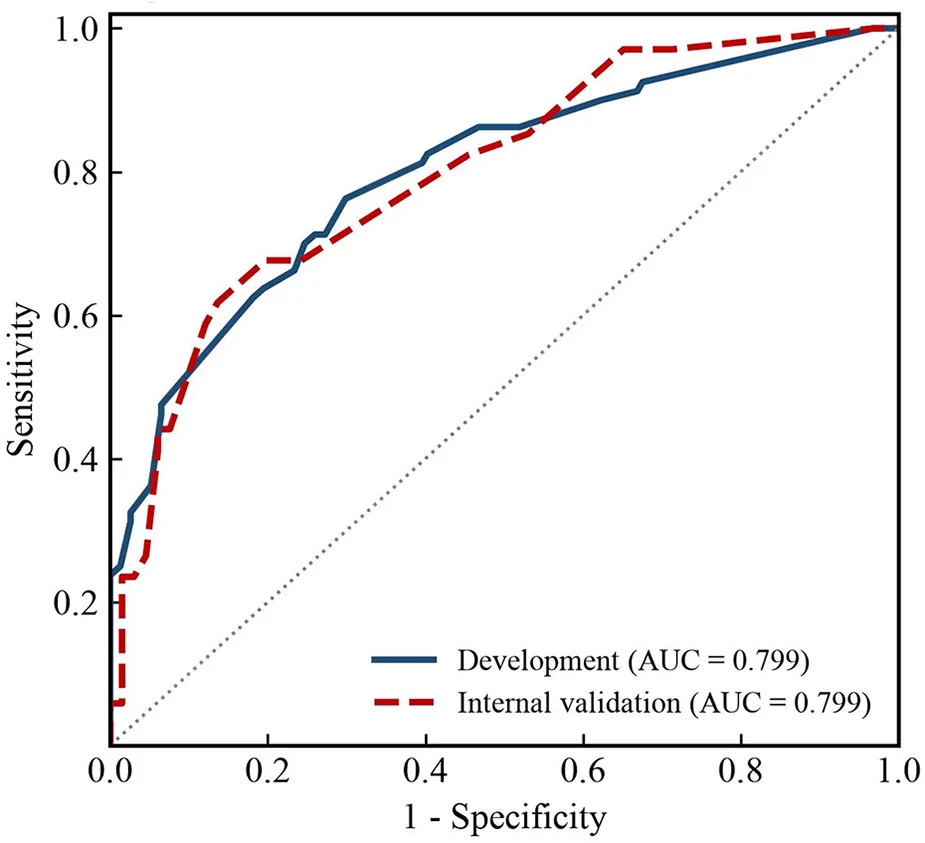
Receiver operating characteristic (ROC) curves of the multivariable model in the development and internal validation cohorts, with bootstrap 95% confidence intervals.

**Figure 4:**
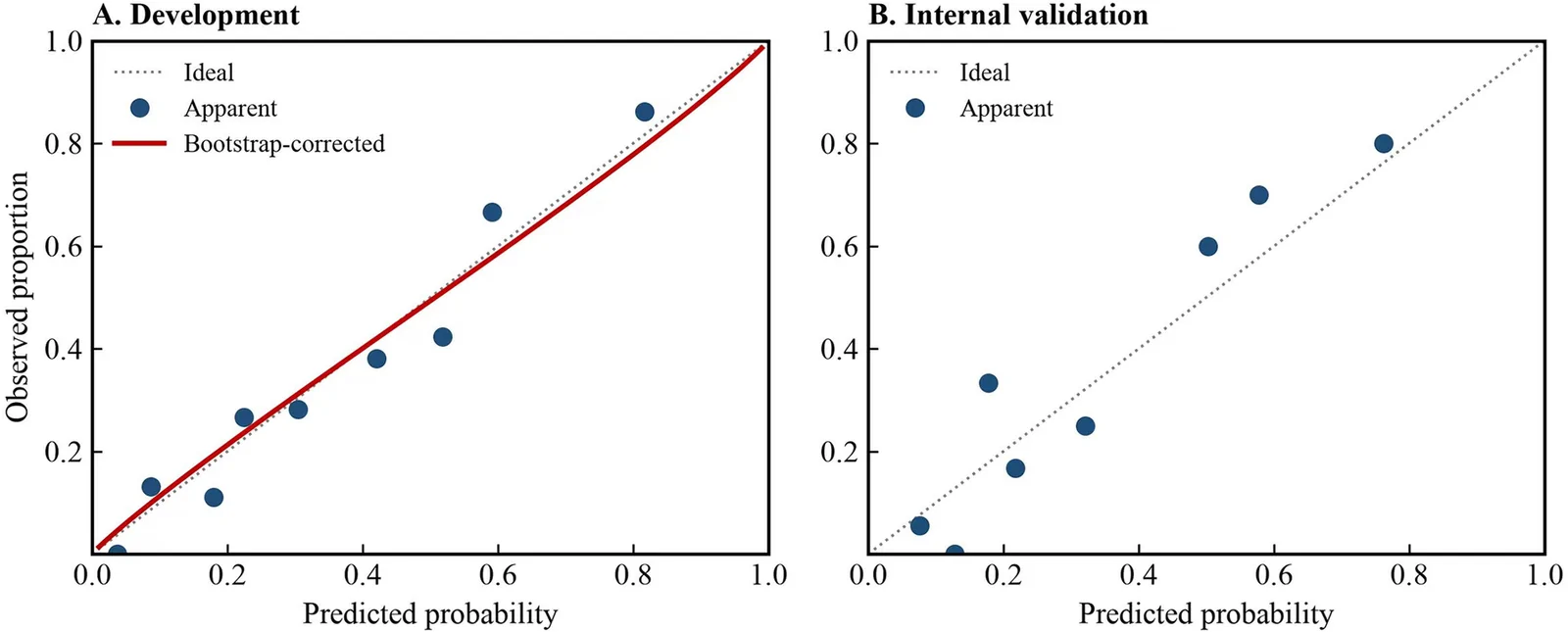
Calibration of the model in the development **(A)** and internal validation **(B)** cohorts; the dashed line indicates perfect calibration and points show grouped observed proportions.

## 4 Discussion

Pulmonary infection is one of the most common diseases affecting older adults. Age-related reductions in the sensitivity of the swallowing and cough reflexes, impaired mucociliary clearance, declining immune function, and a blunted febrile response contribute to the high incidence, severe clinical course, and increased mortality associated with pulmonary infection in this population (21, 22). In addition, older adults commonly have multiple comorbidities and diminished physiological reserve, further contributing to the atypical presentation of pulmonary infection. Because typical respiratory symptoms may be masked by underlying diseases or age-related physiological changes, pulmonary infection may present insidiously in older adults and consequently remain unrecognized (23). In the present study, predictor selection was restricted to a pre-specified candidate set of 14 variables defined *a priori* on the basis of published evidence and clinical plausibility (see Methods, Statistical Analysis). Within this set, backward stepwise selection identified five independent predictors, and LASSO penalized regression, performed as a sensitivity check, retained these five predictors together with three additional candidates, supporting the stability of the selection. Univariable screening was not used to decide candidacy, and candidate variables entered the multivariable model irrespective of their univariate *P* values. Among the haematological markers, substantial multicollinearity was observed among WBC count, neutrophil count, and lymphocyte count, with variance inflation factor (VIF) values of 137.30, 140.23, and 11.43, respectively; WBC count was retained as the most clinically informative and routinely available marker, whereas neutrophil and lymphocyte counts were not entered into the same model in order to avoid multicollinearity and improve model stability. The multivariable analysis showed that age, diabetes mellitus, cerebrovascular disease, decreased appetite, and WBC count were independently associated with occult pneumonia. Altered mental status showed complete separation (present in 9 occult vs. 0 typical patients) and was therefore not retained in the parsimonious model; a Firth penalized logistic regression confirmed that the direction and magnitude of the retained predictors were essentially unchanged (standard-model intercept −4.562 vs. Firth intercept −4.387, with all five coefficients within 7%), supporting the robustness of the final model. These findings suggest that occult pneumonia in older adults is associated with both age-related physiological decline and specific clinical characteristics, including metabolic and cerebrovascular comorbidities, reduced appetite, and abnormal inflammatory markers.

The multivariable model based on the independent factors demonstrated good discrimination in both the development cohort (AUC = 0.799 (95% CI: 0.739–0.854) and the internal validation cohort (AUC = 0.799, 95% CI: 0.699–0.886). The close agreement between the two AUC values suggests that the model had stable discriminative performance and no apparent evidence of substantial overfitting, although external validation is still required. Calibration was further assessed using the calibration intercept, slope, and Brier score, which showed good agreement between predicted and observed probabilities (development: intercept 0.00, slope 1.000, Brier 0.163; internal validation: intercept 0.178, slope 1.185, Brier 0.164). The calibration curves also showed good agreement between the predicted and observed probabilities of occult pneumonia. Among the five predictors, decreased appetite showed the highest standalone discrimination (AUC 0.686 in both the development and internal validation cohorts), followed by diabetes mellitus (0.628 and 0.600), age ≥80 years (0.619 and 0.643), cerebrovascular disease (0.616 and 0.565), and WBC count (0.593 and 0.526); adding the remaining four predictors to decreased appetite increased the AUC to 0.799 in both cohorts, indicating incremental discriminatory value beyond any single predictor. In age-stratified analyses, the model AUC was 0.779 (95% CI: 0.698–0.854) among patients aged 65–80 years (*n* = 178, 47 events) and 0.791 (95% CI: 0.713–0.861) among those older than 80 years (*n* = 122, 61 events), indicating stable discrimination across age strata. Overall, the model showed favorable discrimination and calibration and may assist clinicians in recognizing the occult presentation phenotype among older adults with confirmed CAP, thereby supporting appropriate evaluation and management. Consistent with a graded effect, a continuous-linear sensitivity analysis showed that each 1 × 10⁹/L increase in WBC count was associated with 18% higher odds of occult pneumonia (OR = 1.184, 95% CI: 1.051–1.334; *P* = 0.006), and the restricted cubic spline did not improve fit over the linear form (likelihood-ratio test *P* = 0.920), indicating no threshold or non-linear effect; modelling WBC continuously yielded a model AUC of 0.803 in both cohorts (see Figure 2).

From the perspective of bedside application, the five-predictor model can be reduced to a short checklist that a clinician could carry on a card: (1) age ≥80 years; (2) diabetes mellitus; (3) cerebrovascular disease; (4) decreased appetite; and (5) a WBC count outside the local laboratory reference interval, and particularly above its upper limit. The first four items require only a yes/no answer. The WBC bands used in the model (<3.5, 3.5–9.5, and >9.5 × 10⁹/L) were anchored on the commonly used laboratory reference interval; because the upper and lower limits of normal differ across institutions and assay platforms, we recommend that clinicians applying the model at other institutions substitute the reference interval reported by their own laboratory. In other words, what the model encodes is the direction and grading of the association — progressively higher odds of occult pneumonia as the WBC count rises within and above the reference range — rather than a single universal cutoff.

Advancing age is strongly associated with pneumonia and pneumonia-related mortality. Aging impairs the ability of macrophages to repair tissue damage and regenerate functional tissue in response to inflammatory stimuli, thereby aggravating local lung injury and contributing to immune dysfunction (24, 25). Immunosenescence, dysphagia, a weakened cough reflex, and reduced mucociliary clearance further increase the risk of the occult presentation of pulmonary infection in older adults (26). In addition, reduced respiratory pump function, including decreased respiratory muscle strength and reduced chest wall compliance, together with age-related deterioration in peripheral and central chemoreceptor function, may blunt the ventilatory response to hypoxia (27, 28). Consequently, older adults may develop silent hypoxemia, while early respiratory symptoms during acute illness may be less pronounced.Consistent with previous studies (29, 30), our findings indicate that older adults with occult pneumonia commonly present with fatigue, decreased appetite, and altered mental status rather than prominent respiratory symptoms. Therefore, when an older patient develops unexplained fatigue or decreased appetite, clinicians should consider the possibility of occult pneumonia and initiate timely diagnostic evaluation to avoid delays in treatment. Older adults often have multiple comorbidities and limited organ reserve, making them more vulnerable to systemic decompensation during infection. This may result in nonspecific manifestations such as decreased appetite, fatigue, and altered mental status (31). WBC count is a routinely available marker of systemic inflammation and plays an important role in the clinical assessment of infectious diseases. Previous studies have shown that abnormal WBC counts, including both leukocytosis and leukopenia, are associated with poor outcomes in older adults with community-acquired pneumonia (32, 33). Consistent with these findings (34, 35), our study suggests that abnormal WBC counts may help identify occult pneumonia at an early stage.

Older adults frequently have comorbidities such as diabetes mellitus, cerebrovascular disease, malignant tumors, and conditions requiring immunosuppressive therapy. The combined effects of these conditions may increase the likelihood of an occult presentation of pneumonia, particularly in postoperative or immobilized patients. Previous studies have reported that patients with type 2 diabetes mellitus have a 1.3- to 2.6-fold higher risk of community-acquired pneumonia than the general population, with a further increase in risk among those with poor glycemic control and higher mortality (36, 37). Hyperglycemia and advanced glycation end products may impair innate and adaptive immune function, including neutrophil, monocyte, macrophage, complement, and lymphocyte responses, and may disrupt the pulmonary epithelial–endothelial barrier (38–41). These mechanisms are biologically plausible explanations for the association between diabetes mellitus and occult pneumonia, although they were not directly assessed in the present study. Cerebrovascular disease may also be associated with occult pneumonia. Dysphagia, impaired consciousness, a weakened cough reflex, and prolonged bed rest may increase the risk of aspiration and subsequent pneumonia (42). Dysphagia occurs in approximately 40%–50% of patients after cerebral infarction, and aspiration is associated with an approximately 8- to 11-fold higher risk of pneumonia (43–45). Post-stroke immunosuppression, including lymphocytopenia and impaired natural killer-cell, neutrophil, and monocyte function, has also been proposed as a potential mechanism increasing susceptibility to infection (46, 47). Consistent with previous reports (48, 49), our findings indicate that cerebrovascular disease is independently associated with occult pneumonia. However, stroke timing, dysphagia, and post-stroke immune status were not directly evaluated in this study; therefore, these mechanisms should be regarded as hypotheses. Clinicians should maintain a high index of suspicion in older patients with cerebrovascular disease and strengthen early assessment and monitoring for pneumonia when atypical respiratory symptoms are present.

This study has several limitations. First, it was a retrospective, single-center study based on data from a single department, with a relatively small sample size. Therefore, selection bias may have occurred, and the generalizability of the findings is limited. Second, the number of variables available for analysis was limited, and other potentially important clinical, laboratory, imaging, and functional variables could not be comprehensively assessed. Third, the findings were evaluated only through internal validation. External validation in larger, independent, multicenter cohorts is warranted to assess the stability of the model and its applicability to broader populations. In addition, the WBC bands used in the model were anchored on the reference interval of our central laboratory; institutions with different laboratory reference limits should recalibrate these cut-points to their own intervals when applying the model, and the cross-institutional applicability of the model requires further evaluation. In addition, the diagnostic criteria for occult pneumonia included microbiological indicators, inflammatory biomarkers (PCT and hs-CRP), and treatment response; therefore, verification bias and incorporation bias cannot be completely excluded. Finally, the age threshold of 80 years was primarily based on the age distribution observed in this study and previous literature. Because the model was not separately validated in a narrower age range, such as 65–79 years, its applicability to relatively younger older adults requires further evaluation.

## 5 Conclusions

In conclusion, age, decreased appetite, diabetes mellitus, cerebrovascular disease, and WBC count were independently associated with occult pneumonia in older adults. Because these features are common among older adults with CAP (more than three-quarters of patients in our cohort had at least one), their presence alone is non-specific; nevertheless, clinicians should maintain a high index of suspicion for occult pneumonia when one or more are present—especially in the absence of typical respiratory symptoms—and consider targeted evaluation; a positive screen should prompt, rather than replace, confirmatory assessment. Recognition of occult pneumonia among older adults with CAP may facilitate appropriate clinical management and potentially improve patient outcomes.

## Data Availability

The original contributions presented in the study are included in the article/Supplementary Material, further inquiries can be directed to the corresponding author/s.

## Supplementary material

Data Sheet 1 Raw dataset of the development cohort (n = 234).

Data Sheet 2 Raw dataset of the internal validation cohort (n = 100).

Supplementary Figure S1 STROBE-compliant flow diagram of patient screening, inclusion, and exclusion.
